# Impact of NESA Non-Invasive Neuromodulation on Sleep, Behavior, and Sensory Profile in Children with Autism Spectrum Disorder

**DOI:** 10.3390/children12121599

**Published:** 2025-11-24

**Authors:** Fabiola Molina-Cedrés, Raquel Medina-Ramírez, Aníbal Báez-Suárez, Martín Vílchez-Barrera, Marlene García-Quintana, Andrea Hernandez-Pérez, Irene García-Rodríguez, David Álamo-Arce, Maria del Pilar Etopa-Bitata

**Affiliations:** 1 Sapiens Fisioterapia Clinic, 38430 Tenerife, Spain; fabiola.molina101@alu.ulpgc.es; 2 Soc-Dig Research Group, University of Las Palmas de Gran Canaria, 35016 Las Palmas, Spain; marlenedelcarmen.garcia@ulpgc.es (M.G.-Q.); irene.garcia@ulpgc.es (I.G.-R.); danieldavid.alamo@ulpgc.es (D.Á.-A.); pilar.etopa@ulpgc.es (M.d.P.E.-B.); 3 Faculty of Health Sciences, University of Las Palmas de Gran Canaria, 35016 Las Palmas, Spain; anibal.baez@ulpgc.es (A.B.-S.); martin.vilchez@ulpgc.es (M.V.-B.); andrea.hernandez114@alu.ulpgc.es (A.H.-P.)

**Keywords:** autism spectrum disorder, electrotherapy, sleep quality, neuromodulation

## Abstract

**Highlights:**

**What are the main findings?**

• Non-invasive neuromodulation with NESA microcurrents may produce improvements in sleep quality, aberrant behaviors, and sensory processing in children with autism spectrum disorder.
• The most consistent benefits were observed in sleep resistance, hyperactivity, and sensory hyperreactivity, and some protocols (C and E) showed a greater effect.
• NESA neuromodulation is a non-invasive electrotherapy technique that delivers very low intensity microcurrents through multiple peripheral electrodes on the hands and feet to gently stimulate the autonomic nervous system and promote global neurophysiological regulation.

**What is the implication of the main findings?**

• This pilot series’ cases suggest that neuromodulation targeting the autonomic nervous system may offer a new therapeutic avenue for ASD.
• The results support the feasibility of conducting larger controlled studies in pediatric populations, despite the challenges posed by diagnostic heterogeneity and recruitment.

**Abstract:**

**Background/Objectives**: Autism spectrum disorder is a neurodevelopmental condition affecting up to 1.7% of the global population. Current interventions do not treat the root cause, prompting research into novel treatments like non-invasive neuromodulation. The objective of this study is to examine the use of NESA technology in children with ASD, to determine if it generates changes in their conduct and their central symptoms related to the spectrum. **Methods**: In this study, twelve children with ASD underwent NESA neuromodulation therapy. We assessed the children’s (CSHQ) and parents’ sleep quality (Pittsburg scale), aberrant behavior (ABC-C), and sensory profile (SP-2). **Results**: In most cases (66.7%), we observed an improvement in the test results. A comparative analysis of NESA protocols revealed that the optimal programming strategy involves a longer duration of programs 7 and 8 of NESA microcurrents. **Conclusions**: Given the limited number of patients included in the data set, further investigations are necessary to draw more robust conclusions. This novel form of treatment offers the potential to address autism spectrum disorder by targeting the autonomic nervous system. This approach may influence underlying mechanisms of the autonomic nervous system. The pilot study has opened a new avenue for future research.

## 1. Introduction

Autism spectrum disorder (ASD) is a condition that has seen a significant increase in the number of diagnosed cases in recent years. Its worldwide prevalence is estimated to be between 0.4% and 1.7% of the total population [1,2]. This neurodevelopmental condition is characterized by difficulties in social communication, restricted behaviors and interests, and alterations in the sensorimotor system and stereotypies, among many other indicators [3,4]. Problems in ASD begin to develop in the early stages in life, mainly because language and communication deficits are noticed, for example, in a lack of visual contact, inability to show or understand emotions, or no interest in starting the communication [5]. Sometimes, a language learning regression can even happen [6].

Manifestations of complications in the sensorimotor area cause difficulties in movement and motor attributes, including poor coordination of movement, stereotypies, and affections in gross and fine motor capacity [7,8]. This issue can arise from either excessive or insufficient stimulation within the system [9]. Individuals in the spectrum not only experience difficulties with movement due to altered perception of stimuli, but they experience changes in their senses and emotions too. The atypical motor attributes in these cases may lead to an altered perception of the information received from the senses. This can result in feelings of being overwhelmed by sensory input [10,11].

It is increasingly recognized that motor function is an integral part of the sensory and behavioral phenotype of ASD. Impairments in gross and fine motor coordination, postural control, and oral–motor skills are frequently described and are thought to reflect broader deficits in sensory integration and neural connectivity [12,13,14]. These motor impairments not only affect daily living and communication but also influence how sensory information is processed and responded to, reinforcing the need for interventions that focus on global neurophysiological regulation.

ASD can be combined with various symptoms. One of the most significant associations is with sleep quality. Studies have shown that between 50% and up to 95% of children on the spectrum experience sleep difficulties, which may persist in their adolescence and adulthood [15,16]. Additionally, poor sleep quality has been linked to increased aggressive behavior, impaired social interactions, emotional deficits, and a reduced desire for social interaction in some studies [17]. Moreover, it is important to consider the impact of these factors on families and individuals in the affected environment as well. Over 86% of the parents and families of these individuals also experience poor sleep quality, which can lead to higher rates of anxiety and depression [15,17].

Other facets that are likewise afflicted are problematic behaviors. These may include irritability, hyperactivity, inappropriate language, social withdrawal, or non-compliance, among others [3].

In neuropediatrics, there is no specific treatment for ASD. There are two main types of intervention: biomedical, which involves the prescription of medication, and psychological, which involves a range of different therapies [18].

Medication, physiotherapy, and psychological therapy have demonstrated improvements in symptoms, but these effects do not attend to the root of the disorder itself [19,20]. That is where we can overlap the use of new technologies and new forms of therapy to help these children and, if it is possible, treat the root of the disorder.

The use of non-invasive neuromodulation techniques is emerging as a new treatment option for the nervous system in ASD. Non-invasive brain stimulation (NIBS) has been increasingly investigated as a potential treatment in autism spectrum disorder (ASD), although prior studies have not yet converged on an optimal protocol or target region [21]. Some studies of tDCS and repetitive TMS in individuals with ASD found that NIBS methods could be helpful in ameliorating dimensions such as repetitive behaviors, sociability, and executive/cognitive functions, but cautioned that heterogeneity and publication bias limit strong conclusions [22]. There is some evidence that vagus nerve stimulation (VNS), when performed for epilepsy, may improve behavior in people with ASD, independently of its anti-seizure effects, though higher-quality trials are needed [23]. However, some recent studies with a new technique focused on the autonomic nervous system called non-invasive neuromodulation NESA (Neurostimulación superficial aplicada in Spanish). NESA neuromodulation is a non-invasive electrotherapy technique that applies very low intensity microcurrents through multiple peripheral electrodes placed on the hands and feet to gently stimulate the autonomic nervous system and promote overall neurophysiological regulation, without side effects and proven in studies on cerebral palsy in pediatrics [24]. Previous studies have reported the benefits of this approach in improving sleep quality, reducing constipation, and enhancing cognitive and recovery processes in various neurological and pediatric conditions [24,25,26,27,28]. Although no previous research has examined its application in autism spectrum disorder (ASD), the hypothetical mechanism of action involves the gentle peripheral stimulation of afferent pathways that influence central autonomic and limbic circuits, which play a key role in regulating arousal, emotional control, and sensory processing—areas that are often disrupted in ASD.

Improvements in symptoms related to the autonomous nervous system, such as sleep quality, are produced due to the relationship between the CNS and its implication in the hypothalamus, limbic system (which are superior central regulators of the autonomic nervous system), suprachiasmatic nucleus, and pineal gland. These may be interesting areas of focus for treatments for the symptoms of autism.

The proposed mechanism by which NESA neuromodulation may improve sleep and behavior regulation in autism involves the gentle peripheral modulation of afferent pathways that modulate the central autonomic nervous system. By promoting a balance between sympathetic and parasympathetic activity, this intervention may influence central processes related to arousal, emotion, and sensory integration, systems that are often dysregulated in individuals with ASD.

The objective of this study is to examine the use of NESA neuromodulation in children with ASD to determine if it generates changes in their conducts and their central symptoms related to the spectrum. Specifically, we study the variations in their sensory profile, sleep quality, and behavioral issues.

## 2. Materials and Methods

### 2.1. Subjects

Participants in this study are under-aged individuals who have been clinically diagnosed with autism spectrum disorder. All the children live with their families and are schooled in different types of educational systems. These volunteers are patients in the same physiotherapy clinic, and their parents also participated in this study, providing relevant information as volunteers in this case study.

• Inclusion criteria for participation in this pilot study included the following:
• Being diagnosed with ASD,
• Attending to a non-university educational system.

The exclusion criteria for NESA technology application included the following: having a pacemaker, electricity phobia, or skin injuries, such as eczema or ulcers.

### 2.2. Ethics

In this pilot study, all parents and legal guardians of the subjects were made aware of the conditions of the study, as well as the objectives and treatment that the participants were undergoing. The protocol used was approved by the local ethics committee (see Institutional Review Board Statement). An informed consent document, conducted in accordance with the principles of the Declaration of Helsinki, was signed by all families.

### 2.3. NESA Microcurrents Treatment

**Figure 1:**
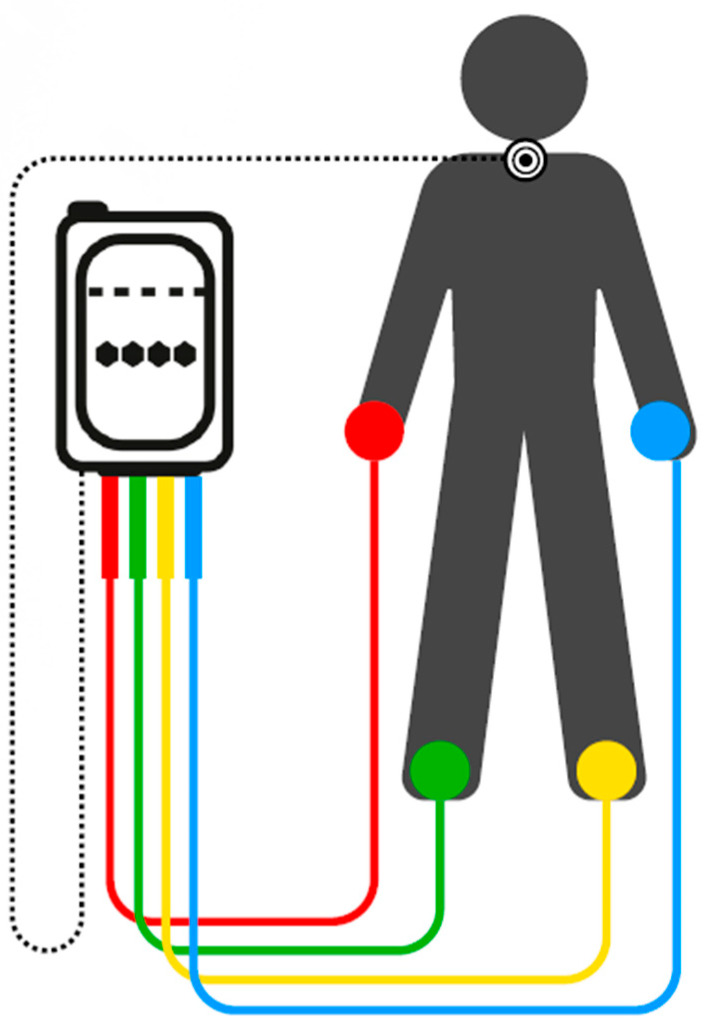
Schematic depicting NESA neuromodulation technology. The colored electrodes are located on the gloves and anklets, with six semi-electrodes on each, and the directional electrode is shown in white in the posterior cervical area.

The treatment employed in this study is based on NESA technology, which is founded upon Wilder’s law and the concept of hormesis. It provokes imperceptible sensations through low impedance areas. The electric current effect is multiplied by its distribution through multiple anatomical pathways due to the electrodes located on the extremities and a directional electrode [26]. The electrodes used by the system are wired into gloves and anklets (or adaptations for amputated limbs), with 25 electrical access points. The current model of the system is minimally invasive and surface-applied (Figure 1). It emits low-frequency pulses ranging from 1.3 Hz to 14.28 Hz, with intensities of 0.1 to 1.0 milliamps and a potential difference of ±3 V and ±6 V, so that the electrical impulse cannot be felt. The impulses are coordinated between 24 electrodes, with 6 electrodes per limb stimulated simultaneously, and a directional electrode [25,27].

To ensure maximum effectiveness, it is necessary to establish a structured and dynamic circuit for the supply of electrical current. This circuit should connect the input points that are jointly connected to the autonomic nervous system and related physiological functions, including the central pathways of the central nervous system (CNS). This can be obtained thanks to the nine different programs that the device offers. Each program employs distinct electrical stimulation at specific points in a predetermined sequence, with a defined duration and polarity change, generated automatically through an algorithm. Following the processing of inputs by the CNS, the autonomic nervous system generates a series of neuromodulated responses. An additional advantage of NESA neuromodulation is its excellent safety and tolerability profile. Unlike transcranial magnetic stimulation (TMS), which can sometimes cause mild adverse effects such as headache, scalp discomfort, or, in rare cases, seizures [29], NESA delivers imperceptible microcurrents through peripheral electrodes without eliciting sensory or muscular responses. No adverse effects have been reported in previous studies using this technology in adults or children, making it particularly suitable for pediatric populations and those with neurodevelopmental disorders [25,26,27].

### 2.4. Intervetion

This study presents a case series in which different types of protocols for the NESA treatment were analyzed (Table 1). We are investigating not only the effectiveness of the NESA technology, but also which one of the proposed protocols is the best within these cases. Every protocol (A–E) mixed different NESA programs (P1, P2, P7, and P8), changing the number of sessions and time as well as the directional electrode placement, whether spinal or sternal. The rationale was to vary treatment intensity by age and logistical constraints, while maintaining exposure to core NESA sequences. These are the characteristics of each protocol:

• Protocol A: Subjects who live near the clinic. Their treatment lasted for 20 sessions, 30 min each. Six participants were treated with this protocol.
• Protocol B: Subjects who live far from the clinic. Their treatment lasted for 15 sessions, 30 min each. Three participants were treated with this protocol.
• Protocol C: Subjects older than 16, with an availability of 3 days a week. Their treatment lasted for 20 sessions, 45 min each. One participant was treated with this protocol.
• Protocol E: Subjects older than 16, without availability. Their treatment lasted for 15 sessions, 45 min each. Two participants were treated with this protocol.

**Table 1:** Protocols description of NESA treatment.

Protocol	Sessions	Duration (min)	Program	Directional Electrode Location
A	1st–5th	15	P1	Spinal C7
		15	P7	
	6th–20th	15	P7	
		15	P8	
B	1st–3rd	15	P1	Spinal C7
		15	P7	
	4th–9th	30	P7	Sternal manubrium
	10th–15th	15	P7	Spinal C7
		15	P8	
C	1st–3rd	15	P1	Spinal C7
		15	P7	
		15	P8	
	4th–9th	15	P2	
		15	P7	
		15	P8	
	10th–15th	30	P7	
		15	P8	
E	1st–3rd	15	P1	Spinal C7
		15	P7	
		15	P8	
	4th–9th	15	P2	Sternal manubrium
		30	P7	
	10th–15th	30	P7	Spinal C7

Table 1 shows the fourdifferent protocols always following the same programs, but with different combinations. The directional electrode means the location of the directional electrode at different body locations.

The programs used are as follows:

- Program 1: Through different polarity impulses, this helps the predisposition to hormesis in the SNA. A minimal dose of application is performed for starting the treatment and generating first contact with the microcurrents.
- Program 2: In this program, microcurrents are directed through the superior members, with an extremely low frequency. This process assists the innervation of nerves in the ventral areas of the organism.
- Program 7: This generates polarity answers that act in the global neuromodulation of the SNA. As a result, an improvement in its functioning is noted, particularly in sleep quality.
- Program 8: The impulses generated influence in different parts of the SNA with the objective of reducing stress and anxiety in the patient.

The placement of electrodes on subjects follows a standardized configuration of NESA technology that uses gloves and anklets with contact points on the hands and feet, along with a mobile directional electrode placed on the cervical region (C7) or the sternal manubrium, depending on the protocol [24]. This arrangement allows for the stimulation of somatic and peripheral autonomic pathways related to the extremities, with the aim of indirectly modulating the central autonomic and limbic circuits involved in the regulation of sleep, emotion, and sensory processing.

### 2.5. Procedure

An appeal was made through a poster to participate in this study of non-invasive neuromodulation NESA for the autistic population. The announcement was disseminated via social media. The patients themselves disseminated the information, and, eventually, 18 individuals from various locations across the island contacted us. Of the 18 individuals who expressed interest, only 13 were able to attend the treatment sessions due to logistical constraints.

Once the families of the participants consented to participate, they were given an initial interview where the procedure to be followed was explained in detail. The objective of the study was presented, which is to improve sleep quality and to observe, in this initial pilot study, the possible changes generated by the treatment. The protocol for the participant with autism was tailored to their age, with the duration of each therapy session adjusted accordingly. The frequency of treatment and number of sessions were also adjusted to align with the family’s logistical constraints and proximity to the center, to minimize disruptions to the participant’s weekly routine.

In this manner, four distinct protocols were established to accommodate the varying requirements in terms of session duration, treatment frequency, and the number of sessions. Once verbal consent had been obtained at the conclusion of the initial interview, the participants were provided with the informed consent document for the study, the data collection form, and the informed consent document for the NESA neuromodulation therapy.

Prior to the inaugural session, the participants were required to complete a series of questionnaires that evaluated the quality of sleep among the individuals and their families, as well as other specific questionnaires about different ASD symptomatology.

The participants were recruited for treatment as they were enrolled in the study. Adaptive equipment was provided for each participant, including gloves and anklets that were tailored to their respective hand and foot sizes. Mats were placed on the floor to accommodate the needs of the smaller participants, who were able to engage in therapeutic activities while seated on them.

At the conclusion of the final session, the data collection questionnaires utilized at the beginning were answered once more, including a novel form pertaining to parental and participant satisfaction with the process undergone.

Of the 13 participants, only one discontinued participation in the study for reasons that remain unknown.

### 2.6. Patient Information

Parents had to complete a demographic questionnaire in which they had to answer questions related to the study variables: age, gender, specific ASD diagnosis, year of diagnosis, who lives at home, whether other therapies are used, type of school, whether the child socializes, level of community support, whether there are comorbidities, and whether medication is used.

### 2.7. Measures

The information collected was derived from the following instruments, which were administered before and after the intervention. All questionnaires were used in their Spanish validated form and were completed by the parents and families of the subjects.

• Children’s Sleep Habits Questionnaire (CSHQ): This instrument permits the examination of sleep disturbances and difficulties in children. The questionnaire is completed by the parents or cohabitants of the children in question and asks about certain circumstances that may indicate sleep difficulties or behaviors related to pediatric sleep difficulties. The response is provided on a Likert scale, and a higher score reflects more troubled sleep, with the maximum score being 99. It employs different subscales, which include sleep resistance, sleep initiation, sleep duration, sleep anxiety, night awakenings, parasomnia, sleep-disordered breathing, and daytime sleepiness [30].
• Sensory Profile (SP-2): This test is a standardized instrument that provides information about the children’s sensory processing patterns in different contexts, including the home, school, and other activities in the community. The test provides an analysis of emotionally self-regulated responses. In this instrument, a higher score (maximum score: 430) reflects a higher reactivity to sensations; therefore, the optimal score would be a lower one [31].
• Aberrant Behavior Checklist (ABC-C). This instrument examines problematic behaviors in patients older than 5 years old with neurodevelopmental disorders. It is answered by families or caregivers of the subject, and it is designed to detect different behavioral problems or symptoms of ASD. It employs a multi-dimensional approach, encompassing a range of subscales, including irritability, self-injurious behaviors, isolation, stereotypies, hyperactivity, and inappropriate language. It is answered with a Likert scale and has a maximum result of 174 points [32].
• Pittsburgh test: This evaluates the quality of sleep of the subjects who respond to the questionnaire. It provides information on both qualitative and quantitative aspects that are correlated with sleep quality over the past month. A score higher than 5 indicates a lower quality of sleep [33]. The test was conducted based on the parents’ responses, to evaluate the sleep quality of families and the treatment’s indirect impact on them.

### 2.8. Statistics

The data were analyzed using the JAMOVI 2.3.21 program. Firstly, the results from all subjects were evaluated without distinguishing between protocols. The scores from the test with information about the subjects were analyzed, followed by the scores from the parents’ and family’s questionnaire. Finally, the different programming protocols were studied. The analysis considers descriptive graphics (means, standard deviations, and case-by-case analyses), considering that one of the protocols only has one study subject. No inferential comparisons were attempted, as the main objective was to explore feasibility and individual responses rather than statistical significance. The case series design was considered appropriate given the heterogeneity of ASD presentations and the well-known challenges of conducting larger controlled trials in this pediatric population.

## 3. Results

### 3.1. Sample

A total of 14 participants initiated the study, of whom 12 (86%) completed all scheduled sessions. The final sample included 12 children and youths with ASD between 4 and 18 years old, mean age—9.4 years, who eventually comprised the study sample. The study had an equal representation of both the sexes. Regarding their schooling, half of them went to special schools, 41.7% attended regular schools, and 8.3% were in special classrooms. Community support was also mainly limited, with 83.3% describing support as insufficient or very insufficient compared with only 16.6% describing it as sufficient or good. Health-wise, 75% had additional medical problems, the most common being sleep disturbances and gastrointestinal complaints. Again, 75% of these participants were under pharmacological treatment, which included stimulants, risperidone, melatonin, and other psychotropic or metabolic agents (see Table S1).

### 3.2. Group Results

When performing descriptive analyses of all cases, in general, the entire sample shows improvements in post-treatment results (see Table 2).

The analysis of the CSHQ indicates a decline in the results following treatment. This phenomenon can be observed in each of the subscales, except for the “sleep-disordered breathing” subscale, which remains unchanged. Upon an examination of the overall scores of the questionnaire, it was observed that the scores showed better results by 7%, which corresponds to 4 points on this instrument.

The ABC-C test showed lower scores after treatment in every subscale examined in the questionnaire. The positive changes in the observed results are evidenced by a decrease in the mean score, which has declined by 12 points (equivalent to 25%). Additionally, the minimum and maximum scores achieved by the subjects indicate a similar trend. The scores decreased by 12 and 29 points, consecutively.

In addition, the SP-2 questionnaire post-treatment score is 12% lower than the pre-treatment score, indicating a positive change in the children’s sensory processing patterns and preferences.

On the other hand, the sleep quality test evaluated in the parents of the subjects was analyzed. Like the children in the study, a decrease in the final score was observed, with a drop of 1.83 points (21% less compared to the initial value) in the families’ scores on the Pittsburgh test. Although the results were not statistically significant, this reduction may reflect a clinically significant change in reported sleep quality.

### 3.3. Comparison Between Treatment Protocols of the Study

Upon examination of each treatment individually for each case, it becomes evident that two distinct forms of programming emerge as the most prominent in terms of results.

**Figure 2:**
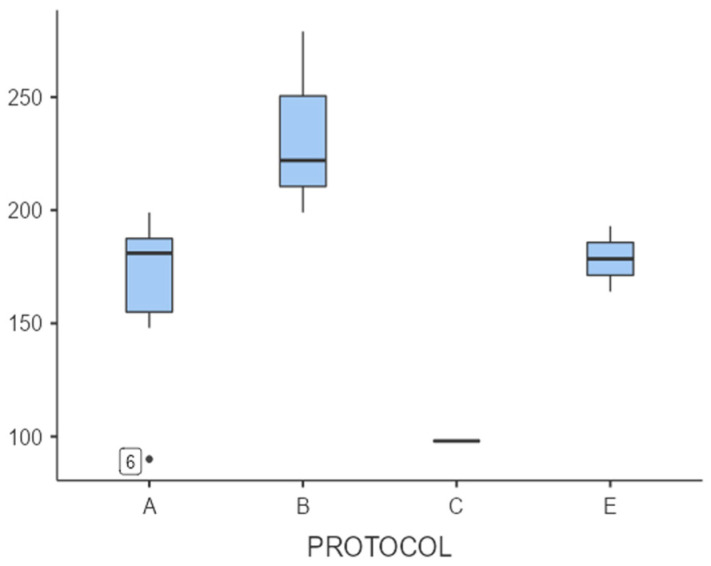
Comparison of scores in SP-2 after treatment between protocols. A box plot comparing the SP-2 results of the different NESA treatment protocols (protocols A, B, C, E) is presented.

**Figure 3:**
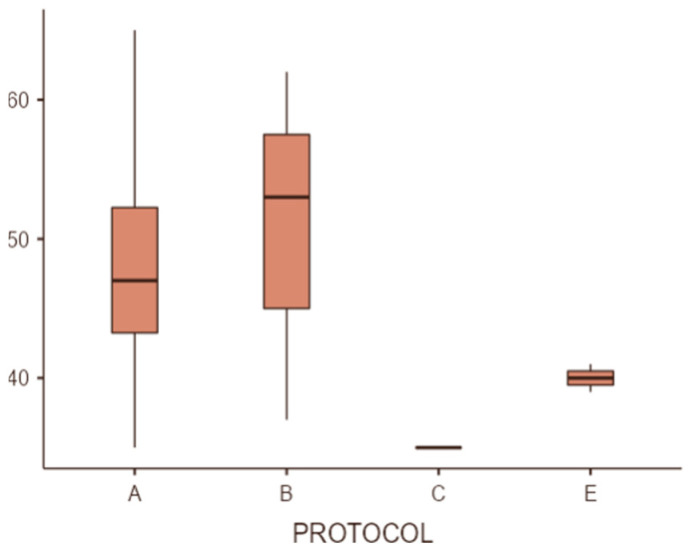
Comparison of scores in CSHQ after treatment between protocols. A box plot comparing the CSHQ results of the different NESA treatment protocols (protocols A, B, C, E) is presented.

**Figure 4:**
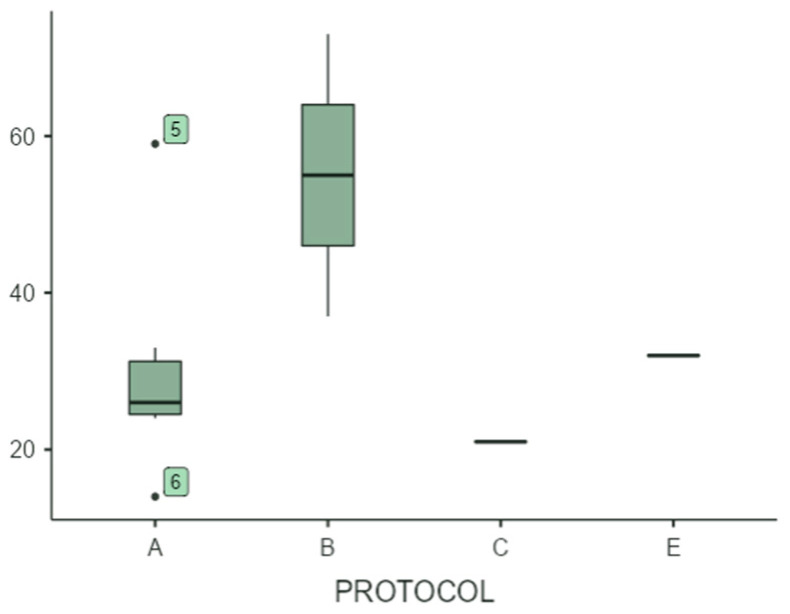
Comparison of scores in ABC-C after treatment between protocols. A box plot comparing the ABC-C results of the different NESA treatment protocols (protocols A, B, C, E) is presented.

Programming E: The results demonstrate a superior performance across all tests, with minimal variability within the group (Figure 2, Figure 3 and Figure 4). This indicates relevant clinical changes in outcomes across participants. The survey data collected after the treatment from the children who underwent this protocol can be observed and compared with the rest of the protocols measured in Table 3. Due to the limited number of participants, the results are not statistically significant. However, they are clinically relevant.

Programming C: Even though only one subject underwent this treatment, it is evident that the most favorable outcomes were observed in all evaluated aspects. The score in all the tests exhibited positive modifications, and these results were superior to those obtained in the other protocols. The most interesting changes are observed in the ABC-C test, which shows a score difference of 9 points or more compared to the other program protocols.

Additionally, we can detect protocol A having satisfactory scores related to the ABC-C, so it must be taken into consideration for future investigations regarding autism and deviant behaviors (Figure 2, Figure 3 and Figure 4).

### 3.4. Qualitative Analysis

The limited number of participants in the study allows for a more granular examination of each case, enabling the observation of subtle differences in treatment effectiveness. In Table 4, a summary of subjects’ pathologies and scores can be found.

The most favorable outcomes of this intervention can be observed in patients 04, 06, 07, 11, and 12, who exhibited positive results and demonstrated improvement in their punctuation across all instruments. Notably, two subjects exhibited the most notable improvement, with patient 11 demonstrating a mean percentage of positive changes of 19% and patient 07 exhibiting a mean of 27% improvement across all instruments. Both patients went under the intervention protocol A. Table 4 presents the percentages changes by subject.

On the other hand, some parents reported no change in the Pittsburgh questionnaire, and in a few cases, an increase in scores was observed in other measures. Nevertheless, overall trends remained favorable, with some degree of progress noted in most participants. Conversely, some patients exhibit a negative response to the intervention, resulting in a decline in their results. These observations are consistent with the case of patients 09, 10, 03, and particularly subject 05. While these results are negative and constitute a retrogression, the percentage of negative results is relatively low (−4.5%).

## 4. Discussion

A total of 14 participants began the study, of whom 12 (86%) completed all scheduled sessions. Dropout was minimal, with only two participants discontinuing for reasons unrelated to the intervention. Attendance was high across all protocols, and no adverse effects or discomfort were reported during NESA sessions, supporting the feasibility and tolerability of the intervention in this pediatric population.

In all sleep-related measures, most participants showed lower scores after the intervention, especially in areas related to difficulty falling asleep and daytime sleepiness. These results suggest that the questionnaires used were sensitive enough to capture intra-individual variations in sleep-related behaviors. In contrast, scores related to sleep-disordered breathing, such as snoring or apnea, remained largely unchanged, which is consistent with expectations for this population. Future studies should explore how these measures can be refined to capture a broader range of sleep patterns in children with ASD.

In terms of behavioral outcomes, several participants showed lower scores on irritability and hyperactivity, while self-injurious behaviors and inappropriate language remained relatively stable. These trends highlight both the variability of individual responses and the feasibility of using parent-reported measures to assess behavioral domains in this context.

Regarding sensory processing, some participants scored lower after treatment, particularly in tactile and socioemotional reactivity. Although these preliminary patterns should not be interpreted as treatment effects, they indicate that SP can detect significant fluctuations within a reasonable time frame, supporting its inclusion in future controlled trials.

Moreover, families may observe relevant changes in themselves throughout the course of the treatment. Cohabitants of the subjects have reported a better sleep quality, leading to an improvement in the families’ sleep patterns and overall quality of life.

As proven in previous studies, protocols that focus on P7 could provide better results for children, especially in their sleep quality [24]. Considering that every ASD case is different and that this is a pilot study with a small sample size, we can predict that the best protocol in ASD treatment is protocol E, in which the directional conductor is changed mid treatment and focuses on P7. The results of the present study, when considered alongside those of the previous one with children with neurodevelopmental disorders, provide a foundation for further research into pediatric neuroscience with NESA microcurrents, with P7 representing a key focus for future protocols [21].

This feasibility study also provided important information for the design of future trials. The small heterogeneity in responses between the different protocols highlights the influence of individual and contextual factors. For example, the participant who underwent Program C, which consisted of longer sessions, showed notable reductions in scores across all domains. This may be related to the longer application of treatment and the subject’s greater ability to participate consistently throughout treatment. These observations highlight the need to adjust session length and individualize schedules in future interventions.

Compared to other treatment methodologies, the combination of pharmacotherapy and behavioral and psychological therapy can yield outcomes on a longer term [34]. Furthermore, these therapies do not target the core symptoms, but rather aim to improve the patients’ social skills, quality of life, and other related issues, such as sleep deprivation, but they could have side effects and adverse reactions in the case of medication [34,35].

Non-invasive brain stimulation (NIBS) techniques, such as tDCS and rTMS, have shown potential benefits in ASD, especially in repetitive behaviors, sociability, and executive functions, although the results are inconsistent and limited by methodological heterogeneity [21,36,37]. Unlike these cortical approaches, NESA neuromodulation acts through multi-electrode peripheral stimulation targeting autonomic regulation, which could explain its effects on both behavior and physiological functions such as sleep and sensory processing. Rather than competing, NESA should be considered a complementary strategy within the field of neuromodulation in ASD.

Lessons learned from this study include the importance of tailoring session length to age, the feasibility of parent reports for outcome collection, and the need to control for individual variability in comorbidities and medication in future trials. NESA neuromodulation may modulate central autonomic regulation, which is implicated in core ASD symptoms. It also could help to predict better results over time, considering that treatment with NESA does not cause dependence due to its own parameters [25].

Despite these promising findings, it is important to consider that, in our study, there are limitations to consider. This study employed a case series format and used four different protocols (in a sample of only 14). It has a small sample that was selected from a single clinic, given the risk of bias, without a comparative control or placebo group. Due to the nature of the study, the only blinding that could be achieved was in the statistical analysis with respect to the type of protocol. Therefore, following this small trial, it is necessary to conduct studies with larger samples, if possible, randomized clinical trials, and even with comparative groups. We encourage other researchers to continue with further research into ASD symptoms.

## 5. Conclusions

This pilot study aimed to explore the effects of the NESA technology on children with ASD, which is a growing problem. As the causes of this disorder are unknown, effective treatment is difficult. The NESA treatment has proven beneficial effects on the children’s symptoms, as well as the quality of sleep in their families. In the future, with a larger sample size, clinical trials, and other age groups, these findings may be positive to keep on studying in further investigation.

## Figures and Tables

**Table 2:** Pre–post treatment scores in CSHQ, ABC-C, SP-2, and Pittsburgh questionnaires.

		Mean	Median	SD	Min	Max
CSHQ	PRE	50.17	51.50	9.13	38.0	66.0
	POST	46.42	43.50	10.18	35.0	65.0
ABC-C	PRE	48.08	39.50	22.85	22	102
	POST	36.00	32.00	17.46	14	73
SP	PRE	203.08	200.00	59.28	105	296
	POST	178.50	187.00	51.03	90	279
Pittsburgh (parents)	PRE	8.75	8.50	4.05	3	18
	POST	6.92	7.00	3.53	1	13

Pre- and post-treatment descriptive variables were determined as a group for an initial descriptive analysis of the sample. SD = standard deviation.

**Table 3:** Comparison of post-treatment mean and DT between protocols.

		Protocol A	Protocol B	Protocol C	Protocol E
CSHQ	Mean	48.3	50.7	35.0	40.0
	SD	10.27	12.66		1.41
ABC-C	Mean	30.3	55.0	21.0	32.0
	SD	15.32	18.00		0
SP-2	Mean	164.5	233.3	98.0	178.5
	SD	40.40	41.19		20.51

SD = standard deviation.

**Table 4:** Individual pre–post scores by subject across all questionnaires.

Subject	Age	Sex	Other Pathologies	Protocol	Assessment Point/Moment	CSHQ	SP-2	ABC-C	Pittsburgh
S01-01	17	F	NOT APPLICABLE	C	1º	42	151	31	7
					2º	35	98	21	7
					IMPROVEMENT	7 (7%)	53 (12%)	10 (6%)	0
S01-02	9	F	SLEEP PROBLEM, HYPERINSULISM	B	1º	66	296	71	8
					2º	53	279	55	10
					**IMPROVEMENT**	**13 (13%)**	**17 (4%)**	**16 (9%)**	**−2 (−9%)**
S01-03	6	F	ADHD	A	1º	41	105	22	9
					2º	50	148	24	7
					**IMPROVEMENT**	**−9 (−9%)**	**−43 (−10%)**	**−2 (−1%)**	**2 (9%)**
S01-04	6	F	ADHD, GASTROINTESTINAL PROBLEMS, SLEEP PROBLEMS	A	1º	54	214	64	18
					2º	53	176	33	13
					**IMPROVEMENT**	**1 (1%)**	**38 (9%)**	**31 (18%)**	**5 (24%)**
S01-05	4	F	INTELLECTUAL DISABILITY, SLEEP PROBLEMS	A	1º	57	186	38	12
					2º	65	199	59	11
					**IMPROVEMENT**	**−8 (−8%)**	**−13 (−3%)**	**−21 (−12%)**	**1 (5%)**
S01-06	7	M	ALERGIES	A	1º	45	142	25	3
					2º	43	90	14	2
					IMPROVEMENT	2 (2%)	52 (12%)	11 (6%)	1 (5%)
S01-07	8	F	NOT APPLICABLE	A	1º	52	285	102	6
					2º	35	188	26	1
					**IMPROVEMENT**	**17 (17%)**	**97 (22%)**	**76 (44%)**	**5 (24%)**
S01-09	4	M	GASTROINTESTINAL PROBLEMS	B	1º	63	231	62	9
					2º	62	222	73	9
					**IMPROVEMENT**	**1 (1%)**	**9 (2%)**	**−11 (−6%)**	**0**
S01-10	5	M	NOT APPLICABLE	B	1º	38	184	45	5
					2º	37	199	37	6
					**IMPROVEMENT**	**1 (1%)**	**−15 (−3%)**	**8 (4%)**	**−1 (−5%)**
S01-11	12	M	SLEEP PROBLEMS	A	1º	53	265	40	13
					2º	44	186	26	4
					**IMPROVEMENT**	**9 (9%)**	**79 (18%)**	**14 (8%)**	**9 (43%)**
S01-12	18	M	NEUROGENIC BLADDER, HYPER-IgE, HYPEREOSINOPHILIC SYND.	E	1º	51	216	38	9
					2º	39	193	32	7
					**IMPROVEMENT**	**12 (12%)**	**23 (5%)**	**6 (3%)**	**2 (9%)**
S01-14	17	M	GASTROINTESTINAL PROBLEMS	E	1º	40	162	39	6
					2º	41	164	32	6
					**IMPROVEMENT**	**−1 (−1%)**	**−2 (−0.5%)**	**7 (4%)**	**0**

The table describes each case with details of age, gender, other comorbidities, the type of NESA treatment protocol applied (A, B, C, E) and the evaluation periods for each of the variables for the subjects and the Pittsburgh for the parents.

## Data Availability

The data supporting the results will be available upon prior request to the authors due to ethical restrictions imposed by Las Palmas Hospital’s Research Ethics Committee.

## Supplementary Materials

The following supporting information can be downloaded at https://doi.org/10.5281/zenodo.17199854 (Created and revised 25 September 2025).

## Abbreviations

The following abbreviations are used in this manuscript:

ASD	Autism spectrum disorder
NESA	Non-invasive neuromodulation system
CNS	Central nervous system
ANS	Autonomic nervous system
CSHQ	Children’s Sleep Habits Questionnaire
SP-2	Sensory Profile, Second Edition
ABC-C	Aberrant Behavior Checklist–Community
SD	Standard deviation
